# Role of computed tomography texture analysis using dual-energy-based bone marrow imaging for multiple myeloma characterization: comparison with histology and established serologic parameters

**DOI:** 10.1007/s00330-020-07320-8

**Published:** 2020-10-03

**Authors:** Christian Philipp Reinert, Eva Krieg, Michael Esser, Konstantin Nikolaou, Hans Bösmüller, Marius Horger

**Affiliations:** 1 Department of Diagnostic and Interventional Radiology, University Hospital Tübingen, Hoppe-Seyler-Str. 3, 72076 Tübingen, Germany; 2 Cluster of Excellence iFIT (EXC 2180) “Image Guided and Functionally Instructed Tumor Therapies”, University of Tübingen, Tübingen, Germany; 3 German Cancer Consortium (DKTK), Partner Site Tübingen, Tübingen, Germany; 4 Institute of Pathology and Neuropathology, University Hospital Tübingen, Liebermeisterstraße 8, 72076 Tübingen, Germany

**Keywords:** Multiple myeloma, Bone marrow, Tumor burden, Image processing, Computer-assisted, Biomarkers

## Abstract

**Objective:**

To identify textural features on dual-energy CT (DECT)–based bone marrow images in myeloma which correlate with serum markers of myeloma activity and the degree of medullary involvement.

**Methods:**

A total of 110 patients (63.0 ± 11.0 years, 51 female) who underwent unenhanced whole-body DECT between September 2015 and February 2019 were retrospectively included, which was approved by our institutional ethics committee with a waiver of the informed consent requirement. All patients had current hematologic laboratory tests. Using DECT post-processing, non-calcium bone marrow images were reconstructed. The vertebral bodies T10–L5 were segmented for quantification of textural features, which were compared with serologic parameters and myeloma stages by the Mann-Whitney *U* test. In a subgroup of 56/110 patients with current bone marrow biopsies, textural features were correlated with the degree of bone marrow infiltration.

**Results:**

First-order features were higher in patients with advanced stage of myeloma (*p* < .02), whereas the 2nd-order “gray-level co-occurrence matrix (GLCM) cluster prominence” was lower (*p* < .04). In patients with elevated serum-free light chains (SFLC) or kappa/lambda SFLC ratio above 1.56, the “entropy” and 2nd-order GLCM features were lower (*p* < .03). The degree of bone marrow infiltration correlated with 1st-order features (e.g., “uniformity”; *r*_P_ = 0.49; *p* < .0001), whereas “entropy” and 2nd-order GLCM features were negatively correlated (e.g., “difference entropy”; *r*_P_ = − 0.54; *p* < .0001).

**Conclusions:**

CT textural features applied on non-calcium bone marrow images correlate well with myeloma-related serologic parameters and histology showing a more uniform tissue structure and higher attenuation with increasing medullary infiltration and could therefore be used as additional imaging biomarkers for non-invasive assessment of medullary involvement.

**Key Points:**

*• Texture analysis applied on dual-energy reconstructed non-calcium bone marrow images provides information about marrow structure and attenuation.*

*• Myeloma-related serologic parameters and the degree of myeloma cell infiltration correlate with 1st- and 2nd-order features which could be useful as additional imaging biomarkers for non-invasive assessment of medullary involvement.*

**Electronic supplementary material:**

The online version of this article (10.1007/s00330-020-07320-8) contains supplementary material, which is available to authorized users.

## Introduction

Multiple myeloma (MM) is a malignant hematologic disease of the mature B cells (plasma cells) which primarily involve the bone marrow [1]. Involvement of the bone marrow is usually assessed by bone marrow biopsy determining the degree of tumor cell infiltration at the initial diagnosis and much rarer later in the course of the disease. By comparison with other hematological diseases, MM is in most cases going along with secretion of paraproteins (e.g., IgG, IgA, light chains), which can be quantified in serum and urine known as the M-gradient and are considered to correlate with the tumor burden [2]. The presence of these tumor markers in most myeloma patients makes their monitoring before, during, and after treatment more reliable compared with other hematologic malignancies. Nevertheless, a minority of MM patients does not secrete these markers and are therefore difficult to surveille by means of laboratory parameters alone which aggravates patient management at primary diagnosis and during therapy [3, 4]. Moreover, myeloma may elude hematologic diagnosis if it expands outside the marrow cavities (extramedullary) [5, 6]. There are in particular these subgroups of MM patients in whom bone marrow imaging is playing a major role as a surrogate to quantify the tumor burden and to monitor the tumor response to anti-myeloma treatment [7]. The most frequently applied imaging techniques for diagnosing MM are X-ray, CT, and MRI [8–10]. The first two imaging modalities have been mainly used for indirect assessment of medullary involvement based on visualization of secondary bone destruction, so-called myeloma bone disease (MBD). In this respect, MR imaging is more sensitive. It is primarily used in most centers and allows for direct assessment of bone marrow changes induced by myeloma cell infiltration [11–14]. MR-based assessment of the extent and pattern of MM mainly operates by evaluation of ancillary T1-weighted and T2-weighted images combined with diffusion-weighted imaging (DWI), where the signal intensity in the bone marrow is compared with the normal marrow of the adults. DWI and perfusion-weighted imaging, which is employed in particular in specialized centers, enable a more accurate assessment of changes occurring at follow-up [15–17]. However, bone marrow signal intensity is also affected by other factors influencing the amount of red and yellow marrow as patient age, treatment-related shifts, or temporal variability which may complicate clinical interpretation [17]. FDG-PET/CT has been lately recommended in treated patients for evaluation of residual myeloma activity [18, 19].

A novel technique called dual-energy CT (DECT) capable of material decomposition and differentiation of different tissue components has been tested for bone marrow imaging. This technique enables to count away the trabecular bone and thus to quantify the bone marrow alone [20–22]. However, this approach is exclusively based on quantification of tissue (bone marrow) attenuation. A novel technique called texture analysis can be applied additionally on these images in order to get more information allowing for more accurate characterization of e.g. tumors focusing on structure, heterogeneity, etc.

Hence, the purpose of this retrospective analysis was to identify CT textural features in the bone marrow of the spine of patients with multiple myeloma which correlate well with established serologic parameters known to reflect tumor activity and histology (degree of myeloma cell infiltration of the bone marrow assessed by biopsy).

## Materials and methods

### Patient characteristics

Our study protocol was approved for retrospective evaluation of patient data by our institutional ethics committee with a waiver of the informed consent requirement (registration number: 019/2019BO2).

All patients with MM who underwent whole-body DECT for staging (no prior therapy) were retrospectively included in this study. Exclusion criteria were a history of other bone disorders and spinal fractures, CT examinations without dual-energy protocol, and no current in-house hematologic laboratory surveillance. Patients who had been currently diagnosed by bone marrow biopsy were separately analyzed. All bone marrow biopsies were performed at the posterior iliac crest. The patients of this subgroup were primarily staged according to the Durie and Salmon classification system [23]. The hematologic diagnoses included the M-gradient as well as serum proteins, serum creatinine, and hemoglobin. All patients regularly (every 3 months) visited our hematology department for follow-up examinations, including laboratory tests and clinical assessment. None of the patients had a hyposecretory or non-secretory multiple myeloma.

### CT examinational protocol

All CT studies were performed using a second-generation DECT scanner (Somatom Definition Flash, Siemens Healthcare). The DECT scanning protocol was standardized dose-reduced and unenhanced as for full-body MM scanning according to our institutional examinational protocols. Tube voltages were set to 100 kV and 140 kV using additional tin filtration for hardening of the high-energy spectrum. Care Dose 4D (automatic exposure control, Siemens Healthcare) was applied. Reference tube current-time products were 230 mAs (100 kV) and 178 mAs (140 kV). Collimation was 64 × 0.6 mm, with a pitch of 0.6 and rotation time of 0.33 s. Total field of view (FOV) was 500 mm; reconstruction FOV was 250 mm. We used an image matrix of 512 × 512. The patient was positioned supine, and the arms were elevated for scanning, ranging from the elbows to the knees, including the complete axial skeleton and the proximal parts of the appendicular skeleton (humeral and femoral bones). DECT image data post-processing was performed on axial and sagittal slices of the entire axial skeleton using a dedicated medium-soft reconstruction kernel (D45f) with a slice thickness of 1.5 mm, an increment of 1.0 mm, and an FOV of 25–30 cm, depending on the patient’s anatomy.

### DECT image evaluation and visualization

Post-processing was performed using approved software for dual-energy data called “dual energy bone marrow” on Syngo.via VB 30A (Siemens Healthineers). The post-processing software is based on a three-material decomposition algorithm. In principle, it is assumed that voxels within the BM can contain three material fractions with different X-ray absorption characteristics (fat, soft tissue, and calcium), which contribute to the total attenuation within the voxel. The total attenuation within the voxel can be separated into a fat and soft tissue partition and a calcium partition which has been already described by Thomas et al [22]. As input data, the prototype uses the low-energy (A series) and high-energy (B series) source data. As an output, two stacks of DICOM images are created: an arithmetic average image of both input series, resembling regular CT images with attenuation (HU) comparable to an image with 120 kV, and a series of virtual non-calcium BM images. Thresholds for the three-material decomposition were set as follows: soft tissue, 57/55 HU (low/high kV); fat, − 103/87 HU; calcium slope, 1.45.

### CT texture analysis of bone marrow images

CTTA was performed using radiomics software (Siemens Healthineers) that is based on the pyradiomics package, a python package for the extraction of radiomics features from medical imaging [24]. A total of 92 original features were analyzed including 1st-order features (*n* = 18), 2nd-order gray-level co-occurrence matrix (GLCM) features (*n* = 23), and the higher order features gray-level dependence matrix (GLDM) (*n* = 14), gray-level run length matrix (GLRLM) (*n* = 16), gray-level size zone matrix (GLSZM) (*n* = 16), and neighboring gray-tone difference matrix (NGTDM) (*n* = 5). Definitions of textural features are listed in Supplementary Table 1.

**Fig. 1:**
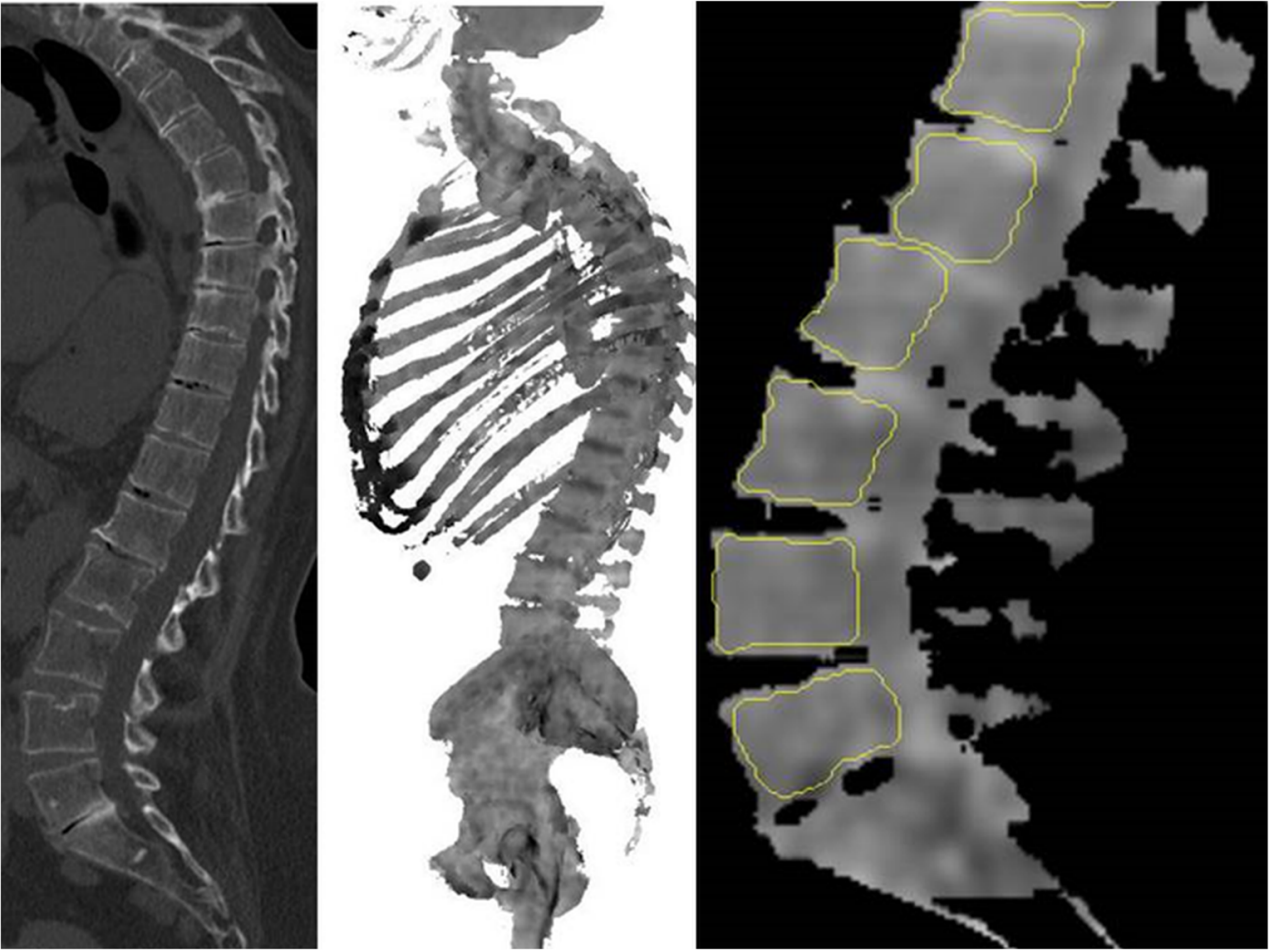
Unenhanced DECT for post-processing and generation of non-calcium “bone marrow image data” which were used for manual segmentation of the vertebral bodies T10–L5. Areas next to the cortical bone, larger bone marrow vessels (e.g., the basivertebral vein and its canal), and focal lytic lesions were excluded

The vertebral bodies T10–L5 were manually segmented using three-dimensional volumes of interest (VOIs) on axial, sagittal, and coronal reconstructed bone marrow CT images. Areas next to the cortical bone, larger bone marrow vessels (e.g., the basivertebral vein and its bony canal) as well as focal lytic lesions, and areas of bony sclerosis were carefully excluded (Fig. 1). Focal lesions which had to be excluded from segmentation are summarized in Table 1. The segmentations were performed by three readers in consensus in a blinded fashion. Two of the readers were radiologists with 30 years and 4 years of experience in diagnostic imaging. For assessment of reliability, segmentations were additionally performed by two readers independently following feature extraction.

**Table 1:** Focal bone lesions excluded from evaluation

Focal lesions	*n*
Sclerotic bone lesions	
Hemangioma	8
Osteochondrosis	45
Enostosis	3
Lytic bone lesions	
Bone cyst	11
Schmorl’s nodes	23

Image filtration was performed for electively extracting features of different sizes and intensity variations followed by quantification of tissue radiomics using series of derived images displaying features at a fine spatial scale (2 mm in radius) within a volume of interest. We performed computation on the current voxels and their neighbors and the results were stored as the texture value of the current voxel. This was repeated for every voxel in the volume of interest.

### Standard of reference

The reference standard was the hematological laboratory including paraproteinemia and paraproteinuria. Due to different thresholds for the serum paraproteins of the myeloma subtypes (e.g., IgA, IgG, IgM), we decided to reduce statistical analysis taking only the serum-free light chain values and their ratios into consideration which are important indicators of myeloma activity [2]. In patients who underwent current biopsy of the bone, the degree of bone marrow infiltration was assessed by our in-house pathologists. Therefore, all specimens are routinely analyzed by two pathologists including a senior pathologist with 30 years of experience to ensure high inter-observer reliability. The mean time interval between DECT and bone marrow biopsy was 5.9 ± 10.9 days (median: 3.0 days, range: 0–48 days).

### Statistical analysis

Statistical analysis was performed using SPSS version 22 (IBM Corporation). We tested all parameters for the normality by the Kolmogorov-Smirnov test. A Mann-Whitney *U* test was used to test the difference in textural features between the different myeloma stages, serologic parameters, and presence or absence of osteolyses. To address the multiple comparisons, a Benjamini-Hochberg correction was applied. The adjusted *p* values were considered significant at a level of 0.05. According to the Guidelines for Reporting Reliability and Agreement Studies (GRRAS) [25], we assessed both the intra- and inter-reader agreement calculating intraclass correlation coefficients (ICCs) from Bland-Altman plots and 95% limits of agreement to determine the reproducibility of our results.

To assess the inter-reader agreement, bone segmentations were performed independently by two readers following feature extraction, whereas the intra-reader agreement was assessed by calculating all textural features twice by the same reader. Reliability coefficients were interpreted as follows: less than 0.50: poor; between 0.50 and 0.75: moderate; between 0.76 and 0.90: good; and over 0.90: excellent [26]. To ensure internal consistency of our data, we performed the split-half reliability test. After splitting all texture data into two equal halves, a Pearson’s *r* correlation was applied. The Pearson’s coefficients were then entered into the Spearman-Brown formula to yield the split-half reliability coefficient.

Furthermore, we applied a z-transformation on all textural features to enable comparability of textural feature values [27, 28]. To measure the strength of the linear relationship between two variables, the Pearson correlation coefficient was calculated and denoted by *r*_P_. A multiple linear regression was calculated to predict the degree of bone marrow infiltration based on textural features. Furthermore, we applied a multivariable logistic regression analysis (forward LR stepwise method) using the most significant textural features to construct multi-indicator models for prediction of myeloma bone disease and pathological/non-pathological serologic markers. To test the significance of the logistic regression model, a *χ*^2^ test was applied, and the Cox and Snell *R*^2^ was calculated.

## Results

### Patient cohort

**Fig. 2:**
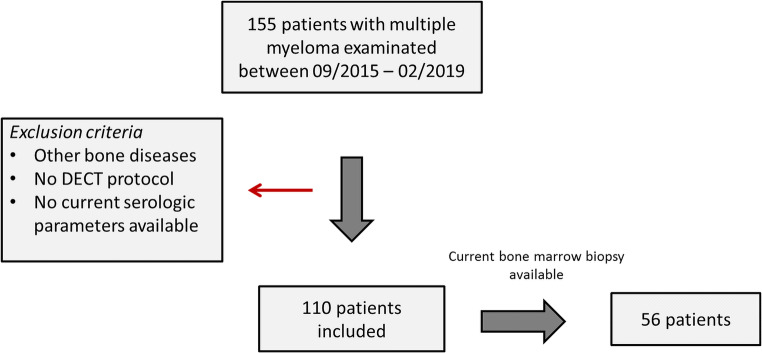
Flow chart of patient selection

A total of 155 patients with MM were referred by the hospital hematology service at our institution to whole-body reduced MDCT in our department. Of these, 20 patients had to be excluded because of a history of cancer with bone metastases, spinal fractures and surgery including osteosynthesis and vertebroplasty, history of radiotherapy, or metabolic disease with bone involvement. Another 25 patients had to be excluded because the CT examination was performed with single-energy and/or no current in-house hematologic laboratory surveillance. Consequently, 110 patients (63.0 ± 11.0 years, 51 female) with MM who underwent whole-body DECT for staging before therapy were included. The clinical characteristics are shown in Table 2. Of this patient cohort, a subgroup of 56/110 patients (63.9 ± 11.8 years, 24 female) who had been currently diagnosed by bone marrow biopsy was separately analyzed (Fig. 2). These patients were primarily staged as stage I (*n* = 16, 28.6%), II (*n* = 10, 17.9%), and III (*n* = 30, 53.6%) MM.

**Table 2:** Patient characteristics

Characteristics	*n*
Age (years)	
Mean ± SD	63.0 ± 11.0
Sex	
Males	59 (53.6%)
Females	51 (46.4%)
Myeloma subtypes	
IgG	56 (50.9%)
IgA	29 (26.4%)
Light chain	25 (22.7%)
Stages	
I	29 (26.4%)
II	21 (19.1%)
III	60 (54.5%)
Kappa/lambda ratio	
Not elevated	53 (48.2%)
Elevated	57 (51.8%)
SFLC	
Not elevated	56 (50.9%)
Elevated	54 (49.1%)
Osteolytic bone lesions	
Yes	39 (35.5%)
No	71 (64.5%)

### Correlation between the degree of bone marrow infiltration and bone marrow textural features

**Fig. 3:**
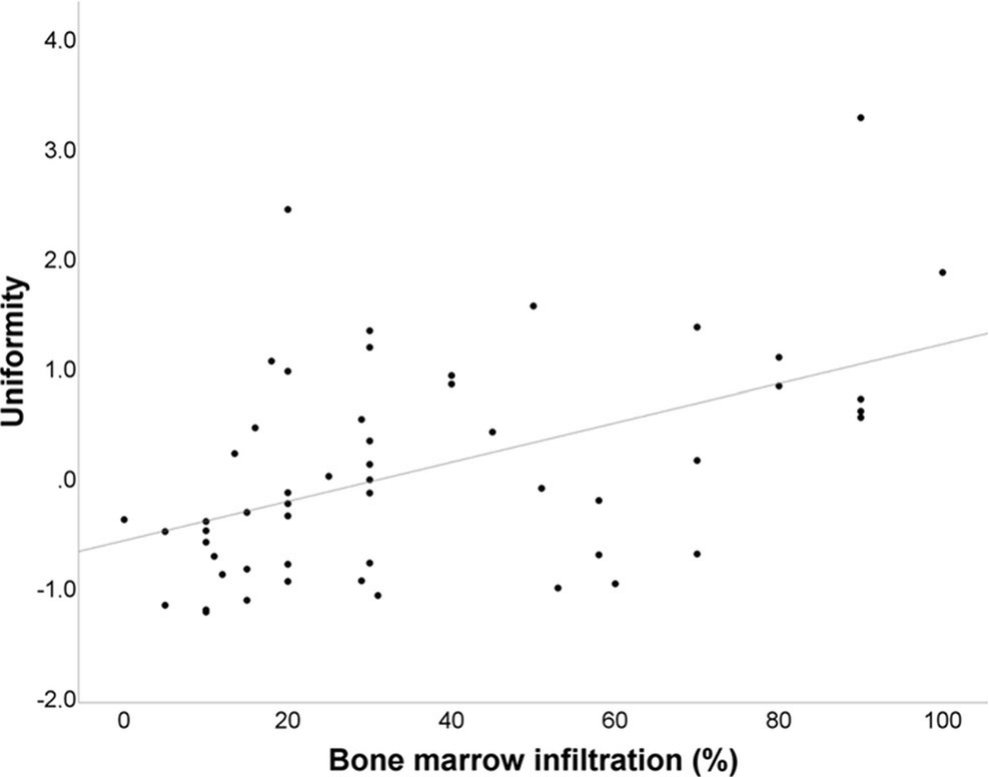
Bivariate correlation curve between bone marrow infiltration (%) and the 1st-order feature “uniformity.” The Pearson’s *r* yielded 0.49 with a *p* value of < 0.0001

**Fig. 4:**
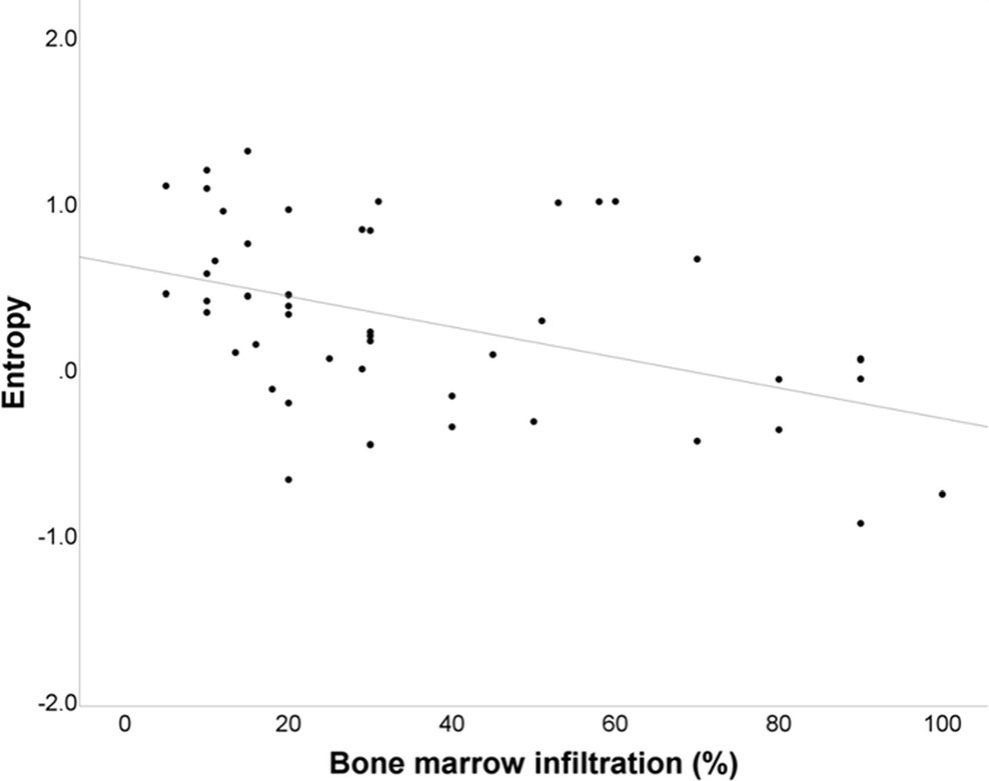
Bivariate correlation curve between bone marrow infiltration (%) and the 1st-order feature “entropy.” The Pearson’s *r* yielded − 0.47 with a *p* value of < 0.001

**Fig. 5:**
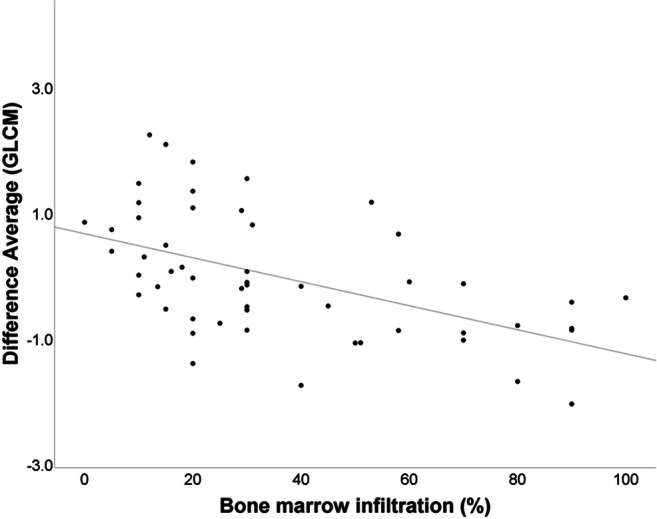
Bivariate correlation curve between bone marrow infiltration (%) and the 2nd-order GLCM feature “difference average.” The Pearson’s *r* yielded − 0.55 with a *p* value of < 0.0001

Absolute values of textural features were correlated with the degree of bone marrow infiltration by bivariate correlation. We observed a significantly positive correlation between the degree of bone marrow infiltration (%) and the 1st-order feature “10th percentile” (*p* < .001) as well as the 1st-order feature “uniformity” (*p* < .0001) (Fig. 3). A significantly negative correlation could be observed between the degree of bone marrow infiltration and the 1st-order feature “entropy” (*p* < .001) (Fig. 4) as well as the 2nd-order GLCM features “contrast” (*p* < .0001), “difference average” (*p* < .0001) (Fig. 5), and “difference entropy” (*p* < .0001).

A significant multiple linear regression equation was found: *F* (6.46) = 4.26, (*p* = 0.002), with *R*^2^ of 0.36. The degree of bone marrow infiltration increased 6.8% for each unit of the 1st-order “10th percentile,” 1.6% for each unit of the 1st-order “uniformity,” and 9.5% for each unit of the 2nd-order GLCM feature “contrast” and decreased 15.7% for each unit of the 2nd-order GLCM feature “difference average,” 4.4% for each unit of the GLCM feature “difference entropy,” and 1.8% for each unit of the 1st order “entropy” (Table 3).

**Table 3:** Multivariate linear regression analysis

Degree of bone marrow infiltration	Pearson’s *r*	*p* value	95% CI	*β*
10th percentile	0.41	< 0.001	− 0.43 to 14.04	6.81
Uniformity	0.49	< 0.0001	− 9.32 to 12.61	1.65
Entropy	− 0.23	< 0.001	− 9.30 to 5.64	− 1.83
GLCM contrast	− 0.50	< 0.0001	− 22.50 to 41.54	9.52
GLCM difference average	− 0.52	< 0.0001	− 56.45 to 25.10	− 15.67
GLCM difference entropy	− 0.52	< 0.0001	− 41.80 to 32.99	− 4.41

*ß*, beta coefficient; *CI*, confidence interval; *GLCM*, gray-level co-occurrence matrix

### Correlation between myeloma bone disease and bone marrow textural features

The 1st-order features “mean” (*p* < .004), “minimum” (*p* < .004), and “10th percentile” (*p* < .003) as well as the higher order feature gray-level run length matrix (GLRLM) “run variance” (*p* < .007) proved all significantly higher in patients presenting with lytic bone lesions compared with patients presenting with no lytic bone lesions.

A logistic regression analysis including these significantly different textural features resulted in a significant model: *χ*^2^ (4) = 31.4; *p* < .001 with *r*^2^_Cox&Snell_ = 0.25, a Nagelkerke’s *r*^2^ = 0.33, and a Cohen’s effect size of *f* = √(0.33/(1–0.33)) = 0.70. In total, 75.5% of patients had been classified correctly as patients with or without myeloma bone disease by the logistic model. The higher order feature GLRLM “run variance” proved to be the variable with the most impact on the odds ratio (Table 4).

**Table 4:** Multivariate logistic regression analysis

	*p* value	Wald	Exp *β*
Myeloma bone disease			
Mean	< 0.004	1.45	1.00
Minimum	< 0.004	2.55	1.02
10th percentile	< 0.003	0.0001	1.00
GLRLM run variance	< 0.007	16.25	1.00
Kappa/lambda ratio			
Minimum	< 0.03	0.61	1.0
GLRLM run variance	< 0.03	2.71	1.0
GLCM sum entropy	< 0.02	6.57	1.0
GLRLM high gray-level run emphasis	< 0.03	3.00	1.0
SFLC			
Entropy	< 0.02	1.71	1.0
GLCM difference average	< 0.03	1.25	< 0.01
GLCM sum entropy	< 0.02	1.92	1.0
GLCM inverse difference	< 0.02	1.15	< 0.01

*Exp ß*, exponentiated beta value; *GLCM*, gray-level co-occurrence matrix; *GLRLM*, gray-level run length matrix; *SFLC*, serum-free light chains

### Correlations between the myeloma stage, the serologic parameters kappa/lambda ratio and SFLC, and the bone marrow textural features

**Fig. 6:**
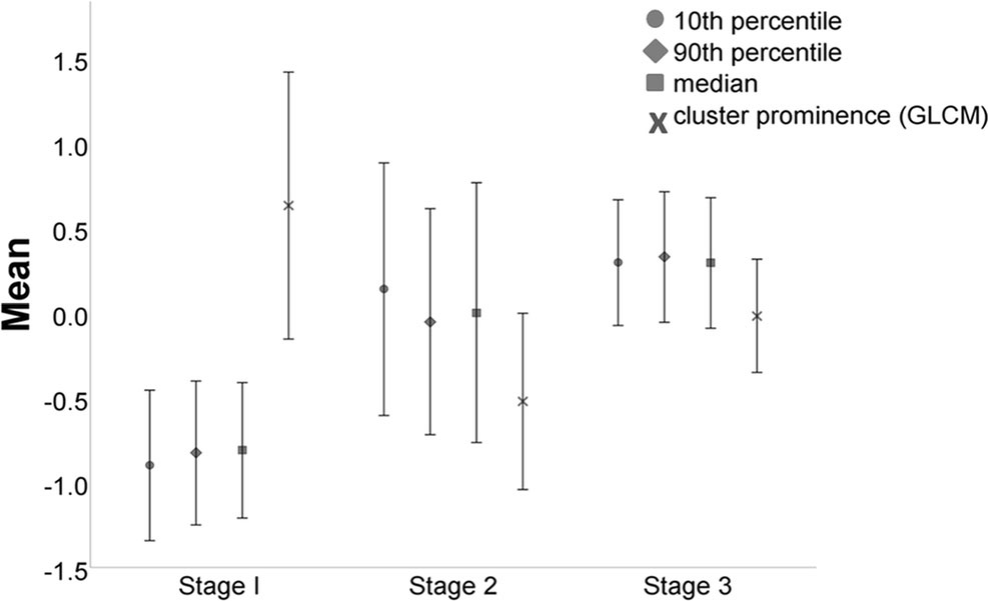
Comparison of the three myeloma stages according to Durie and Salmon classification. The 1st-order features “10th percentile,” “90th percentile,” and “median” were significantly lower in stage I compared with stage 2 or 3 myeloma, whereas the 2nd-order GLCM feature “cluster prominence” was significantly higher (*p* < .05)

Comparing the myeloma stages I–III, we found significant differences for the 1st-order features “10th percentile” (*p* < .01), “90th percentile” (*p* < .01), and “median” (*p* < .02) and the 2nd-order GLCM feature “cluster prominence” (*p* < .04) (Fig. 6).

In patients with pathological kappa/lambda ratio values, the 1st-order feature “minimum” (*p* < .03) and the higher order GLRLM feature “run variance” (*p* < .03) proved significantly higher compared to patients with no pathological kappa/lambda ratio values, whereas the 2nd-order GLCM feature “sum entropy” (*p* < .02) and the higher order GLRLM feature “high gray-level run emphasis” were significantly lower (*p* < .03). The corresponding logistic regression model including these features was *χ*^2^ (4) = 14.6 (*p* = .006) with *r*^2^_Cox&Snell_ = 0.14, a Nagelkerke’s *r*^2^ = 0.18, and a Cohen’s effect size of 0.48. According to this logistic model, 71.0% of patients had been classified correctly as patients with pathological kappa/lambda ratio. The variable with the strongest impact on the odds ratio was the GLCM feature “sum entropy” (Table 4).

Patients presenting with pathologically elevated SFLC showed a significantly lower 1st order “entropy” (*p* < .02), 2nd-order GLCM “difference average” (*p* < .03), and GLCM “sum entropy” (*p* < .02) and a higher GLCM “inverse difference” (*p* < .02) compared with patients in whom the SFLC were not elevated. A logistic regression analysis including these features proved to be significant (*χ*^2^ (4) = 14.4; *p* = .006) with a *r*^2^_Cox&Snell_ of 0.13 and a Nagelkerke’s *r*^2^ of 0.18. The corresponding Cohen’s effect size was 0.48, however, with a lower predictive classification power of this model (66.0% of patients had been classified correctly). The 1st order “entropy” had the most impact on the odds ratio (Table 4).

**Fig. 7:**
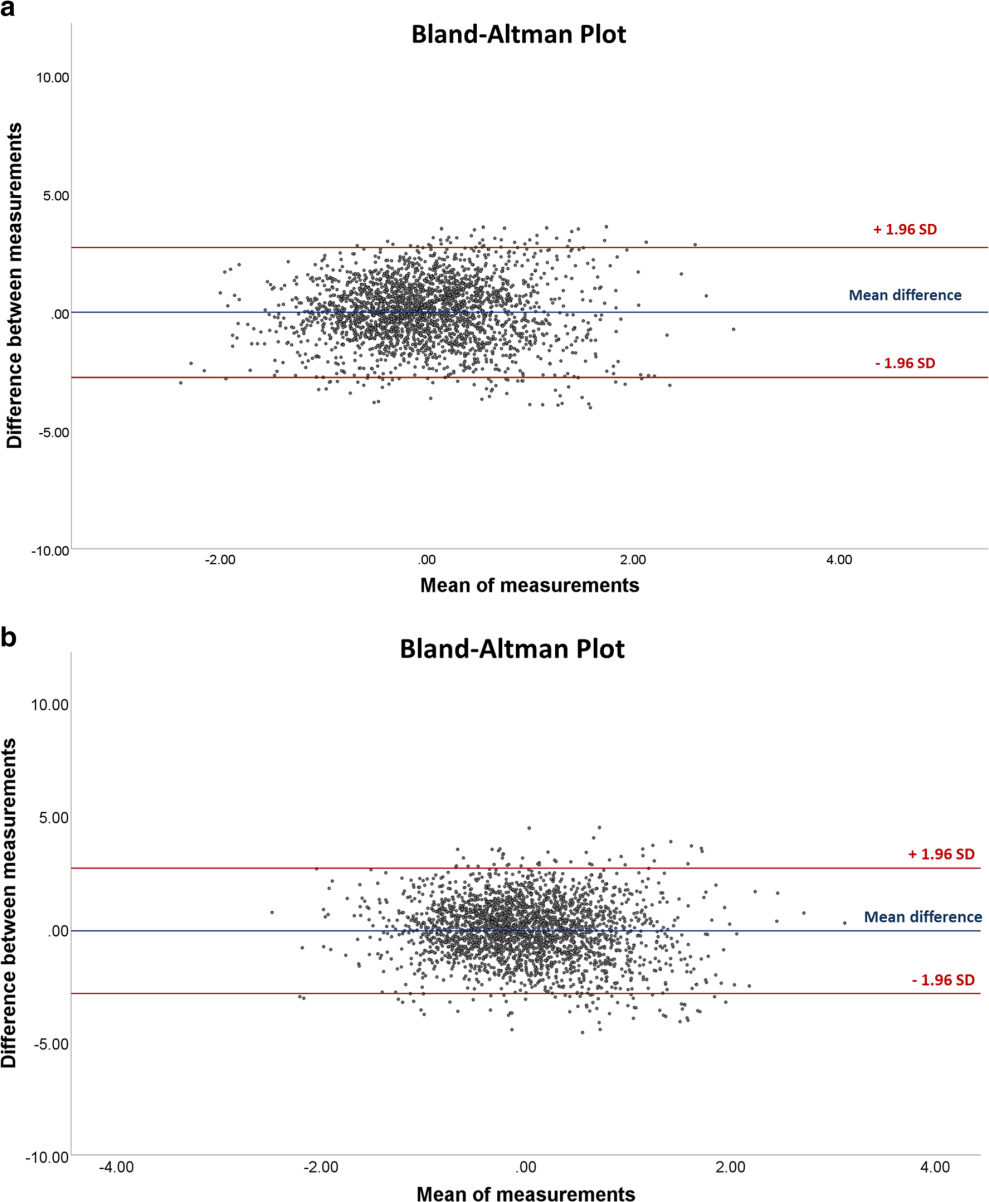
Bland-Altman plots demonstrating the intra-reader reliability (mean difference: − 0.002 ± 1.40) (**a**) and the inter-reader reliability (mean difference: − 0.10 ± 1.41) (**b**)

Regarding the reliability of textural analysis, ICCs ranged from 0.88 (95% CI 0.78–0.96) to 0.95 (0.89–0.98) for intra-reader reliability (Fig. 7a). Results for inter-reader reliability ranged from 0.85 (0.72–0.95) to 0.87 (0.65–0.94) (Fig. 7b). Using the split-half method, the measured textural features had a high internal consistency with calculated Spearman-Brown coefficients above 0.9.

## Discussion

Our results show that there is a strong correlation between textural features quantified on virtual non-contrast unenhanced dual-energy CT images and the degree of myeloma cell infiltration of the bone marrow. We found a significant correlation of medullary involvement with 1st-order features and 2nd-order features with GLCM “difference average” showing the most impact on the multivariate linear regression equation. These parameters reflect an increase in bone marrow attenuation and textural uniformity paralleling the increase in medullary infiltration.

Furthermore, we found that the magnitude of 1st, 2nd, and higher order bone marrow textural features significantly differs in multiple myeloma patients depending on the disease stage as assigned according to the Salmon and Durie classification [23]. Accordingly, the 1st-order features “median,” “10th percentile,” and “90th percentile” significantly increased from stage I to stage III in our patient cohort. These three features are all indicative of increasing bone marrow attenuation accompanying increasing myeloma cell infiltration, proving significantly throughout all stages, but in particular impressively higher in stage III MM. Concomitantly, the 2nd-order feature “cluster prominence” which is a measure of the skewness and asymmetry of the gray-level co-occurrence matrix (GLCM) radically diminished from stage I to stage III MM indicating a more homogeneous bone marrow texture in higher disease stages. Correspondingly, in myeloma patients presenting with lytic bone lesions (myeloma bone disease, MBD), the medullary textural features also significantly differed from those of myeloma patients presenting without MBD. Here again, there were in particular 1st-order features like “mean,” “minimum,” and “10th percentile” that showed higher values in patients with lytic bone lesions compared with patients without MBD. In logistic regression, the 1st order “entropy” had the most impact on the odds ratio. These findings are presumed to reflect higher levels of diffuse myeloma cell infiltration of the bone marrow in patients with MBD. Consequently, the presence and number of lytic bone lesions seem to correlate with the total tumor burden as originally assumed by Salmon and Durie [23]. Moreover, correlation between the degree of myeloma activity based on the levels of serum-free light chains and the textural features of the bone marrow resulted in significant differences. We accordingly observed a lower value of the 1st-order feature “entropy” in patients with elevated SFLC which indirectly indicates a more uniform bone marrow architecture in patients with diffuse myeloma cell infiltration compared to such experiencing reconversion to yellow marrow (e.g., following specific anti-myeloma treatment experiencing tumor resolution) or such who are diagnosed in early stages of the disease. Logistic regression analysis resulted in a significant model showing that the GLCM feature “sum entropy” had the most impact on the odds ratio. In these settings, more structural heterogeneity is expected due to the mixture of hematopoietic cells, marrow adipose tissue, and supportive stromal cells. In patients with inactive disease, conversion to yellow marrow starts early after tumor resolution inducing negative HU values. Myeloma cells have a larger nucleus and a higher nuclear-cytoplasmic ratio and are densely packed, so a more uniform tissue ultrastructure is expected [29]. Nonetheless, this finding is not specific and would be also expected in other disorders going along with increased cell population in the bone marrow. The same trend was also true for the 2nd-order feature “inverse difference” which reflects the local homogeneity of an image on the gray-level co-occurrence matrix. Hence, for more uniform gray levels, the denominator will remain low, resulting in a higher overall value. The 2nd-order feature “difference average” measuring the occurrences of pairs with differing intensity values in the gray-level co-occurrence matrix proved also significantly lower if the SFLC were elevated. Again, the 2nd-order GLCM feature “sum entropy” which is the sum of neighborhood intensity value differences proved significantly lower in patients with elevated SFLC. We obtained similar results while using the kappa/lambda ratios. Here again, the 1st-order feature “minimum” which reflects the lowest attenuation values proved significantly higher in patients with elevated ratios presumed to represent higher and more denser cell populations compared with the yellow bone marrow. The normal kappa/lambda ratio for SFLC is 0.26–1.65 [2]. In multiple myeloma, excessive production of one SFLC type (the clonal component referred to as the involved light chain) often results in an abnormal SFLC ratio [30]. More than 90% of patients with multiple myeloma have altered SFLC ratios that indicate excess production of a clonal FLC by the proliferating plasma cell population [31]. Thus, the SFLC have also an established prognostic value.

Texture analysis has already been applied on spine MRI data of oncologic patients showing potential for discrimination between involved and non-involved bone marrow [32]. Similar to our results, the authors described differences in the 1st-order feature “minimum gray level” on T1w images as well as of 2nd-order GLCM feature “joint variance” which is a measure of heterogeneity highlighting the gray-level variability in tissues [32]. Kawashima et al found that specific textural features derived from regions of interest placed within multiple sites within the skull base and maxillofacial bones can distinguish between patients with normal bone mineral density compared to those with osteoporosis [33]. However, this later approach addressed the bony structures and not the bone marrow.

Our study has some limitations. First, due to the retrospective design, a selection bias cannot be excluded. Several exclusion criteria had to be considered resulting in a limited number of patients. Furthermore, we did not split our study cohort into a training set and a testing set. Second, correlation with histology was possible only for the pelvic bones, but this reflects the usual daily practice in diagnosing bone marrow involvement. Third, as conventional CT is less sensitive for detecting non-lytic diffuse infiltration compared with VNC bone marrow images [22], the obtained HU values should be carefully evaluated as they may differ from the results of the three-dimensional segmentation performed in the VNC images. Therefore, our results should encourage larger studies on this issue to be carried out to establish the real benefit of DE-based CT bone marrow imaging in comparison with conventional CT HU using identical segmentation masks and to identify the most reliable textural features to be implemented for such an approach in a clinical setting. In conclusion, CT textural features applied on non-calcium bone marrow images correlate well with myeloma-related serologic parameters and histology showing a more uniform tissue structure and higher attenuation with increasing medullary infiltration and could therefore be used as additional imaging biomarkers for non-invasive assessment of medullary involvement.

## Electronic supplementary material

ESM 1 (DOCX 56.5 kb)

## Abbreviations

CTTA: CT texture analysis
DECT: Dual-energy CT
DWI: Diffusion-weighted imaging
FOV: Field of view
GLCM: Gray-level co-occurrence matrix
GLDM: Gray-level dependence matrix
GLRLM: Gray-level run length matrix
GLSZM: Gray-level size zone matrix
IgG: Immunoglobulin G
MBD: Myeloma bone disease
MM: Multiple myeloma
NGTDM: Neighboring gray-tone difference matrix
SFLC: Serum-free light chains
