# Supplementary material for: Role of computed tomography texture analysis using dual-energy-based bone marrow imaging for multiple myeloma characterization: comparison with histology and established serologic parameters
Source: Eur Radiol. 2020 Oct 3;31(4):2357–67. doi: 10.1007/s00330-020-07320-8 (PMC7979667; doi:10.1007/s00330-020-07320-8)
Supplement: Supplementary file 1 — (DOCX 56.5 kb) [file 330_2020_7320_MOESM1_ESM.docx]

**Table 1** Definitions of all measured textural features

| Classes | Textural feature | Definition |
| --- | --- | --- |
|  |  |  |
| 1^st^ order  Describe the distribution of voxel intensities within the image region defined by the mask through commonly used and basic metrics. | 10^th^ Percentile | 10^th^ percentile |
|  | 90^th^ Percentile | 90^th^ percentile |
|  | Energy | Measure of the magnitude of voxel values in an image. A larger value implies a greater sum of the squares of these values. |
|  | Entropy | Specifies the uncertainty/randomness in the image values. It measures the average amount of information required to encode the image values. |
|  | Interquartile Range | 25^th^ and 75^th^ percentile of the image array. |
|  | Kurtosis | Measure of the ‘peakedness’ of the distribution of values in the image ROI. A higher kurtosis implies that the mass of the distribution is concentrated towards the tail(s) rather than towards the mean. A lower kurtosis implies the reverse: that the mass of the distribution is concentrated towards a spike near the Mean value. |
|  | Maximum | maximum |
|  | Mean | mean |
|  | Mean Absolute Deviation | Mean distance of all intensity values from the mean value of the image array. |
|  | Median | median |
|  | Minimum | minimum |
|  | Range | Range of gray values in the ROI. |
|  | Robust Mean Absolute Deviation | Mean distance of all intensity values from the mean value calculated on the subset of image array with gray levels in between, or equal to the 10^th^ and 90^th^ percentile. |
|  | Root Mean Squared | Square-root of the mean of all the squared intensity values. It is another measure of the magnitude of the image values. |
|  | Skewness | Measures the asymmetry of the distribution of values about the Mean value. Depending on where the tail is elongated and the mass of the distribution is concentrated, this value can be positive or negative. |
|  | Total Energy | Value of energy feature scaled by the volume of the voxel in cubic mm. |
|  | Uniformity | Measure of the sum of the squares of each intensity value. This is a measure of the homogeneity of the image array, where a greater uniformity implies a greater homogeneity or a smaller range of discrete intensity values. |
|  | Variance | Mean of the squared distances of each intensity value from the mean value. This is a measure of the spread of the distribution about the mean. |
| GLCM  Describes the second order joint probability function for the given region of co-occurring pixel intensities | GLCM Autocorrelation | Measure of the magnitude of the fineness and coarseness of texture. |
|  | GLCM Cluster Prominence | Measure of the skewness and asymmetry of the GLCM. A higher value implies more asymmetry about the mean while a lower value indicates a peak near the mean value and less variation about the mean. |
|  | GLCM Cluster Shade | Measure of the skewness and uniformity of the GLCM. A higher cluster shade implies greater asymmetry about the mean. |
|  | GLCM Cluster Tendency | Measure of groupings of voxels with similar gray-level values. |
|  | GLCM Contrast | Measure of the local intensity variation, favoring values away from the diagonal. A larger value correlates with a greater disparity in intensity values among neighboring voxels. |
|  | GLCM Correlation | Value between 0 (uncorrelated) and 1 (perfectly correlated) showing the linear dependency of gray level values to their respective voxels in the GLCM. |
|  | GLCM Difference Average | Measures the relationship between occurrences of pairs with similar intensity values and occurrences of pairs with differing intensity values. |
|  | GLCM Difference Entropy | Difference Entropy is a measure of the randomness/variability in neighborhood intensity value differences. |
|  | GLCM Difference Variance | Difference Variance is a measure of heterogeneity that places higher weights on differing intensity level pairs that deviate more from the mean. |
|  | GLCM Inverse Difference (ID) | Another measure of the local homogeneity of an image. With more uniform gray levels, the denominator will remain low, resulting in a higher overall value. |
|  | GLCM Inverse Difference Moment (IDM) | Measure of the local homogeneity of an image. IDM weights are the inverse of the Contrast weights (decreasing exponentially from the diagonal i=j in the GLCM). |
|  | GLCM Inverse Difference Moment Normalized (IDMN) | Measure of the local homogeneity of an image. IDMN weights are the inverse of the Contrast weights (decreasing exponentially from the diagonal i=j in the GLCM). Unlike Homogeneity2, IDMN normalizes the square of the difference between neighboring intensity values by dividing over the square of the total number of discrete intensity values. |
|  | GLCM Inverse Difference Normalized (IDN) | Another measure of the local homogeneity of an image. Unlike Inverse Difference, IDN normalizes the difference between the neighboring intensity values by dividing over the total number of discrete intensity values. |
|  | GLCM Informational Measure of Correlation 1 (IMC1) | Assesses the correlation between the probability distributions of i and j (quantifying the complexity of the texture), using mutual information I(x, y). |
|  | GLCM Informational Measure of Correlation 2 (IMC2) | IMC 2 also assesses the correlation between the probability distributions of i and j (quantifying the complexity of the texture). |
|  | GLCM Inverse Variance | Inverse Variance of GLCM. |
|  | GLCM Joint Average | Joint average returns the mean gray level intensity of the i distribution. |
|  | GLCM Joint Energy | Measure of homogeneous patterns in the image. A greater Energy implies that there are more instances of intensity value pairs in the image that neighbor each other at higher frequencies. |
|  | GLCM Joint Entropy | Measure of the randomness/variability in neighborhood intensity values. |
|  | GLCM Maximum Probability | Maximum Probability is occurrences of the most predominant pair of neighboring intensity values. |
|  | GLCM Sum Average | Measures the relationship between occurrences of pairs with lower intensity values and occurrences of pairs with higher intensity values. |
|  | GLCM Sum Entropy | Sum of neighborhood intensity value differences. |
|  | GLCM Sum Squares | Sum of Squares or Variance is a measure in the distribution of neigboring intensity level pairs about the mean intensity level in the GLCM. |
| GLDM  Quantifies gray level dependencies in an image. A gray level dependency is defined as the number of connected voxels within distance δ that are dependent on the center voxel. | GLDM Dependence Entropy | Measures the entropy in dependence size in the image. |
|  | GLDM Dependence Non Uniformity | Measures the similarity of dependence throughout the image, with a lower value indicating more homogeneity among dependencies in the image. |
|  | GLDM Dependence Non Uniformity Normalized | Measures the similarity of dependence throughout the image, with a lower value indicating more homogeneity among dependencies in the image. |
|  | GLDM Dependence Variance | Measures the variance in dependence size in the image. |
|  | GLDM Gray Level Non Uniformity | Measures the similarity of gray-level intensity values in the image, where a lower GLN value correlates with a greater similarity in intensity values. |
|  | GLDM Gray Level Variance | Measures the variance in grey level in the image. |
|  | GLDM High Gray Level Emphasis | Measures the distribution of the higher gray-level values, with a higher value indicating a greater concentration of high gray-level values in the image. |
|  | GLDM Large Dependence Emphasis | Measure of the distribution of large dependencies, with a greater value indicative of larger dependence and more homogeneous textures. |
|  | GLDM Large Dependence High Gray Level Emphasis | Measures the joint distribution of large dependence with higher gray-level values. |
|  | GLDM Large Dependence Low Gray Level Emphasis | Measures the joint distribution of large dependence with lower gray-level values. |
|  | GLDM Low Gray Level Emphasis | Measures the distribution of low gray-level values, with a higher value indicating a greater concentration of low gray-level values in the image. |
|  | GLDM Small Dependence Emphasis | Measure of the distribution of small dependencies, with a greater value indicative of smaller dependence and less homogeneous textures. |
|  | GLDM Small Dependence High Gray Level Emphasis | Measures the joint distribution of small dependence with higher gray-level values. |
|  | GLDM Small Dependence Low Gray Level Emphasis | Measures the joint distribution of small dependence with lower gray-level values. |
| GLRLM  Quantifies gray level runs, which are defined as the length in number of pixels, of consecutive pixels that have the same gray level value. | GLRLM Gray Level Non Uniformity | Measures the similarity of gray-level intensity values in the image, where a lower GLN value correlates with a greater similarity in intensity values. |
|  | GLRLM Gray Level Non Uniformity Normalized | Measures the similarity of gray-level intensity values in the image, where a lower GLNN value correlates with a greater similarity in intensity values. |
|  | GLRLM Gray Level Variance | Measures the variance in gray level intensity for the runs. |
|  | GLRLM High Gray Level Run Emphasis | Measures the distribution of the higher gray-level values, with a higher value indicating a greater concentration of high gray-level values in the image. |
|  | GLRLM Long Run Emphasis | Measure of the distribution of long run lengths, with a greater value indicative of longer run lengths and more coarse structural textures. |
|  | GLRLM Long Run High Gray Level Emphasis | Measures the joint distribution of long run lengths with higher gray-level values. |
|  | GLRLM Long Run Low Gray Level Emphasis | Measures the joint distribution of long run lengths with lower gray-level values. |
|  | GLRLM Low Gray Level Run Emphasis | Measures the distribution of low gray-level values, with a higher value indicating a greater concentration of low gray-level values in the image. |
|  | GLRLM Run Entropy | Measures the uncertainty/randomness in the distribution of run lengths and gray levels. A higher value indicates more heterogeneity in the texture patterns. |
|  | GLRLM Run Length Non Uniformity | Measures the similarity of run lengths throughout the image, with a lower value indicating more homogeneity among run lengths in the image. |
|  | GLRLM Run Length Non Uniformity Normalized | Measures the similarity of run lengths throughout the image, with a lower value indicating more homogeneity among run lengths in the image. |
|  | GLRLM Run Percentage | Measures the coarseness of the texture by taking the ratio of number of runs and number of voxels in the ROI. |
|  | GLRLM Run Variance | Measure of the variance in runs for the run lengths. |
|  | GLRLM Short Run Emphasis | Measure of the distribution of short run lengths, with a greater value indicative of shorter run lengths and more fine textural textures. |
|  | GLRLM Short Run High Gray Level Emphasis | Measures the joint distribution of shorter run lengths with higher gray-level values. |
|  | GLRLM Short Run Low Gray Level Emphasis | Measures the joint distribution of shorter run lengths with lower gray-level values. |
| GLSZM  quantifies gray level zones in an image. A gray level zone is defined as the number of connected voxels that share the same gray level intensity. A voxel is considered connected if the distance is 1 according to the infinity norm (26-connected region in a 3D, 8-connected region in 2D). | GLSZM Gray Level Non Uniformity | Measures the variability of gray-level intensity values in the image, with a lower value indicating more homogeneity in intensity values. |
|  | GLSZM Gray Level Non Uniformity Normalized | Measures the variability of gray-level intensity values in the image, with a lower value indicating a greater similarity in intensity values. |
|  | GLSZM Gray Level Variance | measures the variance in gray level intensities for the zones. |
|  | GLSZM High Gray Level Zone Emphasis | Measures the distribution of the higher gray-level values, with a higher value indicating a greater proportion of higher gray-level values and size zones in the image. |
|  | GLSZM Large Area Emphasis | Measure of the distribution of large area size zones, with a greater value indicative of more larger size zones and more coarse textures. |
|  | GLSZM Large Area High Gray Level Emphasis | Measures the proportion in the image of the joint distribution of larger size zones with higher gray-level values. |
|  | GLSZM Large Area Low Gray Level Emphasis | Measures the proportion in the image of the joint distribution of larger size zones with lower gray-level values. |
|  | GLSZM Low Gray Level Zone Emphasis | Measures the distribution of lower gray-level size zones, with a higher value indicating a greater proportion of lower gray-level values and size zones in the image. |
|  | GLSZM Size Zone Non Uniformity | Size-Zone Non-Uniformity (SZN) measures the variability of size zone volumes in the image, with a lower value indicating more homogeneity in size zone volumes. |
|  | GLSZM Size Zone Non Uniformity Normalized | Measures the variability of size zone volumes throughout the image, with a lower value indicating more homogeneity among zone size volumes in the image. |
|  | GLSZM Small Area Emphasis | Measure of the distribution of small size zones, with a greater value indicative of more smaller size zones and more fine textures. |
|  | GLSZM Small Area High Gray Level Emphasis | Measures the proportion in the image of the joint distribution of smaller size zones with higher gray-level values. |
|  | GLSZM Small Area Low Gray Level Emphasis | Measures the proportion in the image of the joint distribution of smaller size zones with lower gray-level values. |
|  | GLSZM Zone Entropy | Measures the uncertainty/randomness in the distribution of zone sizes and gray levels. A higher value indicates more heterogeneneity in the texture patterns. |
|  | GLSZM Zone Percentage | Measures the coarseness of the texture by taking the ratio of number of zones and number of voxels in the ROI. |
|  | GLSZM Zone Variance | Measures the variance in zone size volumes for the zones. |
| NGTDM  Quantifies the difference between a gray value and the average gray value of its neighbours within distance δ. | NGTDM Busyness | Busyness is a measure of the change from a pixel to its neighbour. A high value for busyness indicates a ‘busy’ image, with rapid changes of intensity between pixels and its neighbourhood. |
|  | NGTDM Coarseness | Coarseness is a measure of average difference between the center voxel and its neighbourhood and is an indication of the spatial rate of change. A higher value indicates a lower spatial change rate and a locally more uniform texture. |
|  | NGTDM Complexity | Complexity: An image is considered complex when there are many primitive components in the image, i.e. the image is non-uniform and there are many rapid changes in gray level intensity. |
|  | NGTDM Contrast | Contrast is a measure of the spatial intensity change, but is also dependent on the overall gray level dynamic range. Contrast is high when both the dynamic range and the spatial change rate are high, i.e. an image with a large range of gray levels, with large changes between voxels and their neighbourhood. |
|  | NGTDM Strength | Strenght is a measure of the primitives in an image. Its value is high when the primitives are easily defined and visible, i.e. an image with slow change in intensity but more large coarse differences in gray level intensities. |

GLCM = Gray Level Co-occurrence Matrix; GLDM = Gray level Dependence Matrix; GLRLM = Gray Level Run Length

Matrix; GLSZM = Gray Level Size Zone Matrix; NGTDM = Neighbouring Gray Tone Difference Matrix
